# Characterization of adipocytes derived from fibro/adipogenic progenitors resident in human skeletal muscle

**DOI:** 10.1038/cddis.2015.79

**Published:** 2015-04-23

**Authors:** N Arrighi, C Moratal, N Clément, S Giorgetti-Peraldi, P Peraldi, A Loubat, J-Y Kurzenne, C Dani, A Chopard, C A Dechesne

**Affiliations:** 1 UFR Sciences, Université Nice Sophia Antipolis, Nice F-06108, France; 2 CNRS, UMR7277, F-06108 Nice, France; 3 INSERM U1091, F-06108 Nice, France; 4 INSERM U 1065, Mediterranean Research Centre for Molecular Medicine, Team: Cellular and Molecular Physiopathology of Obesity and Diabetes, Nice, France; 5 Hôpitaux Pédiatriques de Nice CHU-Lenval, Nice, France

## Abstract

A population of fibro/adipogenic but non-myogenic progenitors located between skeletal muscle fibers was recently discovered. The aim of this study was to determine the extent to which these progenitors differentiate into fully functional adipocytes. The characterization of muscle progenitor-derived adipocytes is a central issue in understanding muscle homeostasis. They are considered as being the cellular origin of intermuscular adipose tissue that develops in several pathophysiological situations. Here fibro/adipogenic progenitors were isolated from a panel of 15 human muscle biopsies on the basis of the specific cell-surface immunophenotype CD15^+^/PDGFR*α*^+^CD56^−^. This allowed investigations of their differentiation into adipocytes and the cellular functions of terminally differentiated adipocytes. Adipogenic differentiation was found to be regulated by the same effectors as those regulating differentiation of progenitors derived from white subcutaneous adipose tissue. Similarly, basic adipocyte functions, such as triglyceride synthesis and lipolysis occurred at levels similar to those observed with subcutaneous adipose tissue progenitor-derived adipocytes. However, muscle progenitor-derived adipocytes were found to be insensitive to insulin-induced glucose uptake, in association with the impairment of phosphorylation of key insulin-signaling effectors. Our findings indicate that muscle adipogenic progenitors give rise to *bona fide* white adipocytes that have the unexpected feature of being insulin-resistant.

Adipose tissue consists of several distinct anatomical compartments. It is not completely clear how all of them are formed despite the current high interest in understanding adipose tissue specificities. One intriguing compartment is the so-called intermuscular adipose tissue (IMAT), which can be found between muscle fibers.^1, 2^ Adipocytes accumulate and replace a large proportion of muscle fibers in muscular dystrophies,^3^ and muscle adiposity was even shown to be an accurate measurement of the severity of Duchenne muscular dystrophy.^4^ IMAT accumulation has also been reported in type II diabetes,^5, 6^ aged muscles,^7, 8, 9^ denervation,^10^ and in chronic disuse-induced muscle atrophy.^11, 12^ IMAT accumulation also occurs in muscles of healthy younger individuals after only 4 weeks of immobilization.^11^ In pathological and nonpathological models, IMAT accumulation is linked to insulin resistance.^5, 13, 14^ The fat infiltration of muscle has not been markedly investigated for many years, whereas this process likely has deep impacts on muscle function because of the profound alterations induced in muscle structure and the important interplay between muscle and adipose tissues – which are both known to be very active factor-secreting tissues.^15^

Muscle regeneration is supported by the extensively characterized satellite cells, which are myogenic progenitors laying along muscle fibers.^16^ In addition, a few groups have recently identified adipogenic progenitors resident in skeletal muscle. Among them are progenitors identified on the basis of specific cell-surface marker expressions, which can thus be physically separated by cell sorting. In humans, muscle adipogenic progenitors have been separated by flow cytometry as a CD15^+^CD56^−^ subpopulation by us and others.^17, 18, 19, 20^ CD56, the neural cell adhesion molecule 1, is known to be expressed by muscle satellite cells (which have the CD15^−^CD56^+^ immunophenotype). CD15 is an antigenic carbohydrate molecule found in several glycoproteins. Before its implication in the muscle adipogenic lineage, it was essentially known to be present in hematopoietic and neural cells. The CD15^+^CD56^−^ adipogenic progenitors express the mesenchymal stem or progenitor cell markers CD13, CD34, CD44, CD49, CD90, and CD105. They are negative for the lineage markers CD31, CD45, CD106, CD117, CD133, and STRO-1.^18, 19, 20^

In parallel in mice, muscle fibro/adipogenic progenitors (FAPs) have been identified as lin^−^(*α*7 integrin)^−^Sca-1^+^CD34^+^ cells^21^ and muscle mesenchymal progenitors with the immunophenotype CD31^−^CD45^−^SM/C-2.6^−^PDGFR*α*^+^ have been shown to contribute to fat cell formation in skeletal muscle.^22^ Further studies indicated that the two mouse immunophenotypes in fact specifically label the same progenitors that should be recognized as skeletal muscle-resident mesenchymal progenitors.^23^ Finally, PDGFR*α* has also been used very recently in human to isolate muscle mesenchymal progenitors, which are equivalent to the mouse FAPs.^24, 25^

Despite the physiological importance of adipocytes derived from human or mouse skeletal muscle, characterization of these terminally differentiated cells is essentially limited to the expression of adipogenic markers. No comprehensive analyses have been reported, and the extent to which muscle adipogenic progenitors differentiate into fully functional adipocytes is unknown.

Here we benefited from the recent identification of these progenitors to investigate their differentiation, as well as the functional characteristics and specificities of the derived adipocytes. The whole study has been performed in humans considering the functional importance of human IMAT. Muscle biopsies were taken from a panel of 15 donors. Canonical adipose stroma cells (ASCs) prepared from subcutaneous adipose tissue depots, and their derived adipocytes were used as references. In this study, we established first that the PDGFR*α*^+^CD56^−^ muscle progenitors are identical to the CD15^+^CD56^−^ progenitors, which therefore, can be also considered as the human counterparts of the FAPs isolated in mice. Then, our cellular, molecular, and biochemical data showed that *bona fide* white adipocytes are derived from human muscle-resident progenitors. However, these adipocytes have an unexpected impairment in insulin signaling associated with insulin resistance with reduced glucose uptake.

## Results

### CD15 and PDGFR*α* specifically label the same human muscle FAP subpopulation

**Figure 1:**
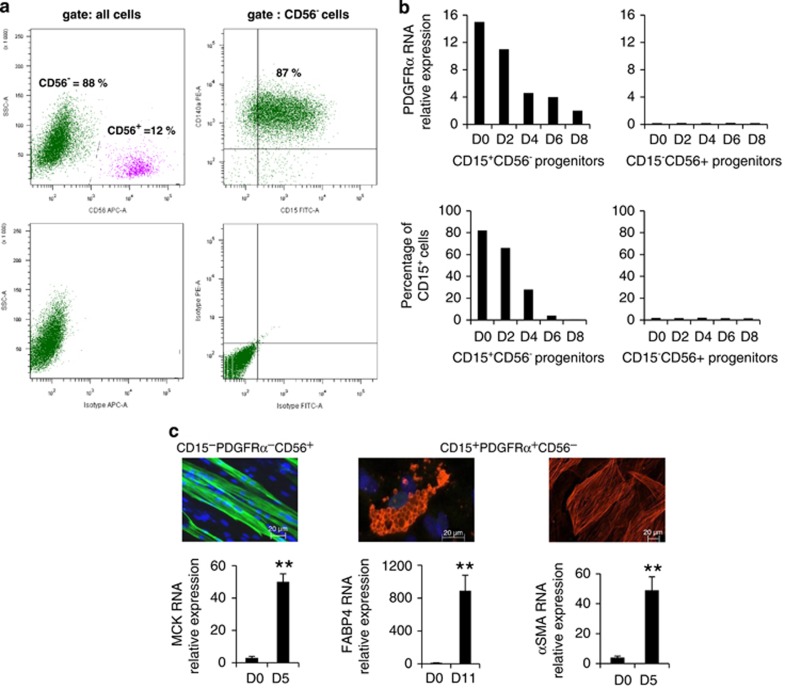
CD15 and PDGFR*α* are specific markers of human FAPs. (**a**) Representative results from flow cytometry analysis of adherent human muscle cells. The CD56 negative cells (left upper panel) were labeled with both anti-CD15 and anti-PDGFR*α* (CD140a; right upper panel). The lower panels represent the respective isotype negative labelings. Percentages of the fractions are indicated. (**b**) Kinetic expression of PDGFR*α* and CD15 shown for a representative muscle biopsy (FAP biopsy 7). PDGFR*α* expression was measured by quantitative RT-PCR and CD15 expression was measured by flow cytometry. At day D0, the cells were confluent and proliferation medium was replaced by differentiation medium. Measurements were done every 2 days until day D8. (**c**) After confluence (D0), cells were put under differentiation conditions. Myotubes were labeled with antimyosin heavy-chain antibodies and muscle creatine kinase (MCK) expression was compared with quantitative RT-PCR between days D0 and D5. Adipocytes were stained with oil red O and FABP4 expression was compared with quantitative RT-PCR between days D0 and D11. Fibroblast-like cells were labeled with anti-*α* smooth muscle actin and its expression was compared with quantitative RT-PCR between days D0 and D5. Quantitative RT-PCR results are mean±S.E. of the mean (*n*=3; FAP biopsies 2, 9, and 10). **P<0.01. FITC, fluorescein isothiocyanate; PE, phycoerythrin; SSC, side scatter detector

After culture expansion on a plastic support, muscle adipogenic progenitors were sorted by flow cytometry on the basis of the positive expression of CD15 and the negative expression of the myogenic marker CD56. As already observed, the proportions of CD15^+^CD56^−^ and CD15^−^CD56^+^ cells were biopsy dependent. To compare the CD15^+^CD56^−^ cell subpopulations with the PDGFR*α*^+^ CD56^−^ FAPs reported by Uezumi *et al.*,^24^ we analyzed CD56^−^ muscle-derived cells with both anti-CD15 and anti-PDGFR*α* (also named CD140a) antibodies. Figure 1a shows that almost all CD56^−^ cells correspond to a single population that is positive for both CD15 and PDGFR*α* (88±9% *n*=6). Expression of PDGFR*α* measured by quantitative RT-PCR decreased along differentiation of CD15^+^CD56^−^ adipogenic progenitors, whereas it was not found in CD15^−^CD56^+^ myogenic progenitors (Figure 1b). The expression of CD15, measured by flow cytometry, followed the same pattern. We compared the differentiation potentials of cell subpopulations sorted according to CD15, PDGFR*α*, and CD56 expressions, and submitted to the appropriate differentiation medium. Differentiated cells were characterized by their phenotypes and the expression of specific markers assessed with immunofluorescence or quantitative RT-PCR (Figure 1c). No differences were seen between the CD15^+^ and PDGFR*α*^+^ cells. The CD15^−^/PDGFR*α*^−^CD56^+^ cells differentiated only into myotubes and the CD15^+^/PDGFR*α*^+^CD56^−^ cells differentiate into adipocytes or fibroblast-like cells, but not into myotubes. Together, CD15 and PDGFR*α* label the same human muscle-proliferating progenitors, which have a fibro/adipogenic potential without myogenic potential. Anti-CD15 and anti-PDGFR*α* antibodies can be alternatively used to sort human muscle FAPs.

### Time course of adipogenic differentiation and adipocyte phenotype

**Figure 2:**
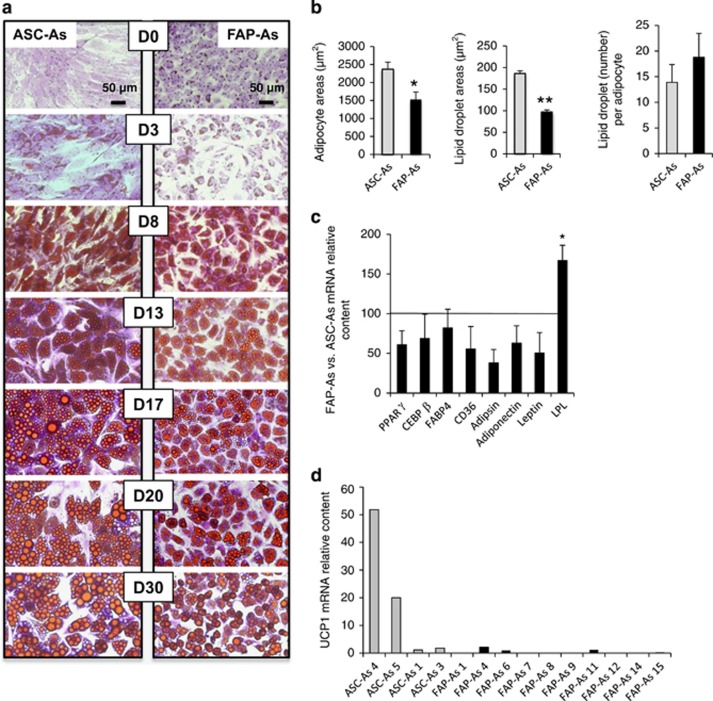
Adipogenic differentiation. (**a**) FAPs and ASCs were grown to confluence in adjacent culture wells and then treated with differentiation-inducing medium. Cells were fixed and assayed for intracellular lipid droplets with oil red O staining from day 0 (start of differentiation induction) to day 30, as indicated. Cell cultures were counterstained with crystal violet. Pictures were visualized by light microscopy with × 200 magnification. Fields representative of whole cell culture wells are shown and were obtained with ASC biopsy 4 and FAP biopsy 6. Scale bar=50 *μ*m. (**b**) Adipocyte and lipid droplet sizes (*μ*m^2^) were analyzed using ImageJ software in cultures after 2 weeks of adipogenic differentiation. Lipid droplet quantity was estimated by the intracellular droplet number counted per adipocyte. ASC-As are represented in gray bars (*n*=3; ASCs biopsies 1, 3, and 4) and FAP-As in black bars (*n*=3; FAP biopsies 4, 6, and 7). Results are mean±S.E. of the mean for three independent measurements. *P<0.05, **P<0.01. (**c**) FAP-As mRNA content of classical early (PPAR*γ* and CEBP*β*) and late (FABP4, CD36, adipsin, adiponectin, leptin, and LPL) adipogenic markers was quantified by quantitative RT-PCR. The results are expressed as ratios (%) to respective ASC-As mRNA contents, which are represented by the horizontal line. FAP-As (*n*=3; FAP biopsies 4, 6, and 7) and ASC-As (*n*=3; ASC biopsies 1, 2, and 4) were strictly maintained under the same culture conditions. Results are mean±S.E. of the mean for three independent ratio measurements *P<0.05. (**d**) UCP1 expression was measured with quantitative RT-PCR in FAP-As, which were prepared from a panel of biopsies (*n*=10; FAP biopsies 1, 4, 6, 7, 8, 9, 11, 12, 14, and 15) in comparison with ASCs-As from biopsies 1, 3, and 4. *P<0.05, **P<0.01

Muscle FAPs and ASCs were submitted to the same adipogenic differentiation conditions. Throughout differentiation, they both exhibited very close morphological appearances, characteristic of adipocytes with the cytoplasm accumulating growing lipid droplets (Figure 2a). Hereafter, adipocytes derived from FAPs will be designated as FAP-As and adipocytes derived from ASCs as ASC-As. A similar time course of adipogenic differentiation was observed for the two cell types, and oil red O-triglyceride-specific staining was obtained from day 3 of differentiation. A canonical image of *in vitro* fully mature adipocytes was noted after about 2 weeks of differentiation and was preserved for several weeks. This could be observed up to 80 days (Supplementary Figure 2). FAP-As as well as ASC-As were filled by a decreasing number of larger and larger lipid droplets. However, adipocyte areas and lipid droplet areas were significantly reduced in FAP-As (Figure 2b). The number of lipid droplets remained equally distributed in both types of adipocytes.

Therefore, muscle FAPs differentiate into adipocytes under the same culture conditions as ASCs, with the same kinetics and acquire a very close phenotype. We only noticed a smaller size of adipocytes associated with smaller lipid droplets for FAP-As.

### Induction and inhibition of adipogenic differentiation

The expression of early and late main canonical markers of adipocyte differentiation was examined with quantitative RT-PCR to assess the molecular mechanisms involved in adipogenic differentiation. No significant difference was found between FAP-As and ASC-As for the expression levels of peroxisome proliferator-activated receptor (PPAR)*γ*, CEBP*β*, FABP4, CD36, adipsin, adiponectin, and leptin (Figure 2c). This quantitative result is in accordance with the oil red O staining shown in Figure 2a. Unexpectedly, lipoprotein lipase (LPL) was found to be significantly more expressed in FAP-As. Expression of the early markers PPAR*γ* and CEBP*β* was detected right at the beginning of differentiation, whereas terminal markers such as FABP4 were detected only at day 3 of differentiation (Supplementary Figure 3). This was similar for FAP-As and ASC-As. Thus, muscle FAPs gave rise to adipocytes according to a differentiation pattern very similar if not identical to that of ASCs in terms of adipogenic marker identity, kinetics, and expression level.

As muscle FAPs were found to follow the same differentiation pathways as ASCs, we wondered whether the pathways known for inhibiting adipogenesis of adipose tissue progenitors were also functional in muscle adipogenic progenitors. We treated these progenitors with TNF*α* or cytokines from the transforming growth factor (TGF)*β* family, that is, TGF*β*1 and activin A^26, 27, 28^ (Supplementary Figure 4). These factors drastically inhibited differentiation of muscle FAPs, with no or only very little adipocyte formation. This showed that adipogenesis is negatively regulated in FAPs by same crucial molecules as those involved for ASCs.

### Muscle FAP cells differentiate into white adipocytes

We investigated the white or brown nature of FAP-As, as brown adipocyte progenitors have also been described in human muscle.^29^ The expression of uncoupling protein one (UCP1), which is the major brown adipocyte marker, was assessed in FAP-As prepared from muscle samples obtained from 10 donors. A lack of expression or only a very weak expression was found (Figure 2d). The expression was always much lower than UCP1 expression found in adipocytes derived from some of the ASCs samples. Note that FAP-As included young and adult donors, which showed that the lack of UCP1 expression could not be ascribed to an age effect.

### Triglyceride synthesis, storage, and lysis

As adipogenic activities have been found to be age dependent,^30, 31^ FAP-As derived from donors <9 years of age were used to match ages of ASC references.

**Figure 3:**
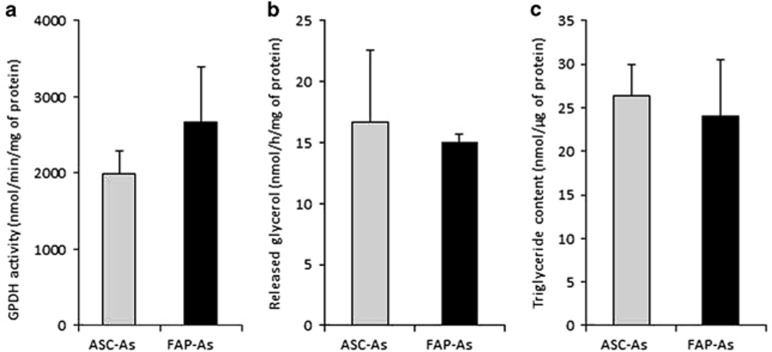
Triglyceride synthesis, storage, and lysis. Biochemical activities were measured in FAP-As and ASC-As prepared from age-matching donors (<9 years). (**a**) Triglyceride synthesis was assessed with GPDH activity. (**b**) Lipolysis was assayed by glycerol released. (**c**) Lipid storage was estimated through triglyceride content measurement. ASC-As values are represented with gray bars (*n*=3; ASC biopsies 1, 2, and 4), FAP-As values in black bars (*n*=3; FAP biopsies 4, 6, and 7). Results are mean±S.E. of the mean for three independent measurements. No significant differences were found between ASC-As and FAP-As

Triglyceride biosynthesis was assayed with glycerol-3-phosphate dehydrogenase (GPDH) activity and no significant difference was observed between FAP-As and ASC-As (Figure 3a). Lipolysis, that is, triglyceride degradation, was assayed by glycerol release induced with forskolin, used as a nonselective *β*-adrenoreceptor agonist (Figure 3b). FAP-As had also an activity similar to ASC-As. Finally, the ability to accumulate intracellular lipids was measured by the triglyceride content. Once more, no difference was observed between the two types of adipocytes (Figure 3c).

Regarding the effect of age, GPDH activity, amount of released glycerol, and triglyceride content were altered in adipocytes derived from adult FAP donors (from 14 to 59 years of age) as compared with young FAP donors (less than 9 years of age; data not shown). These biochemical activities indicate that FAP-As fulfill the fundamental lipid-related functions of adipocytes.

### Impaired glucose transport

**Figure 4:**
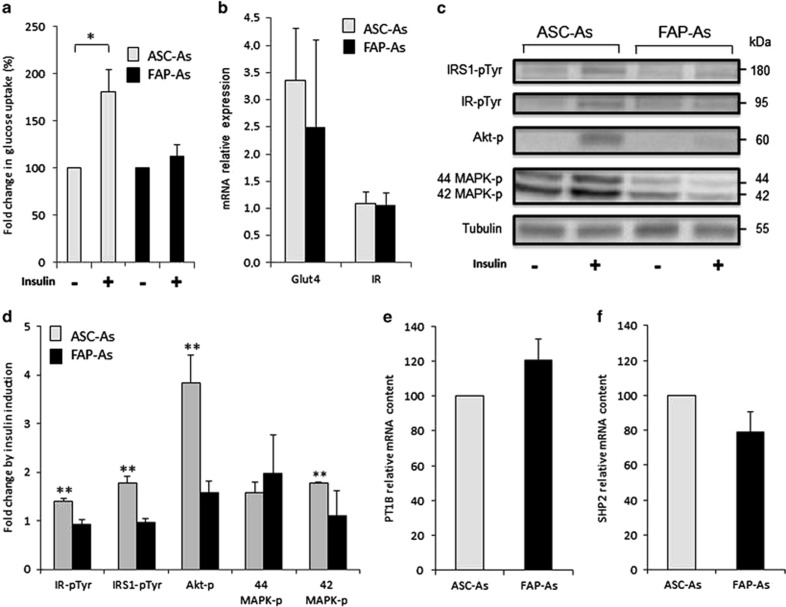
Impaired glucose transport and alterations in insulin signaling. (**a**) Insulin-stimulated glucose transport was determined using nonmetabolizable [^3^H]2-deoxy-d-glucose. The results are expressed as a percentage of glucose transport measured in the absence of insulin separately for ASC-As and FAP-As. ASC-As values are represented with gray bars (*n*=4; ASC biopsies 1, 2, 3, and 4) and FAP-As values with black bars (*n*=8; FAP biopsies 3, 4, 5, 6, 7, 13, 14, and 15). (**b**) mRNA relative expression of Glut4 and IR measured by quantitative RT-PCR. *n*=3 for ASC-As (ASC biopsies 1, 3, and 4) and FAP-As (FAP biopsies 4, 6, and 7). The results are mean±S.E. of the mean for three independent measurements. (**c**) Phosphorylations were compared in adipocytes without insulin treatment and with 100 nM insulin stimulation, for 20 min. Cell lysates were subjected to immunoblotting with specific antibodies. Representative blots are shown. (**d**) Immunoblot signals were quantified and normalized with tubulin signals. Phosphorylated-form contents are expressed as fold change induced by insulin relative to the absence of insulin treatment. The phosphorylations concern IR tyrosines, IRS-1 tyrosines, Akt-Thr^308^, 42 MAPK-Thr^202^, and 44 MAPK-Tyr^204^. ASC-As values (*n*=3; ASC biopsies 1, 2, and 4) are represented with gray bars and FAP-As values in black bars (*n*=6; FAP biopsies 1, 2, 8, 9, 10, and 12; except for 42 and 44 MAPK where *n*=4; FAP biopsies 1, 2, 8, and 9). Data are expressed as mean±S.E. of the mean. (**e** and **f**) Relative mRNA contents of PTP1B and SHP2 were measured by quantitative RT-PCR and expressed as a percentage of values found in ASC-As. For ASC-As, *n*=5 (ASC biopsies 1, 2, 3, 4, and 5); for FAP-As, *n*=7 (FAP biopsies 2, 4, 6, 8, 9, 10, and 12). The results are mean±S.E. of the mean for three independent measurements. *P<0.05 and **P<0.01

One key feature of adipocytes is that they are insulin-sensitive cells. Insulin induces translocation of the glucose transporter Glut4 to the plasma membrane, resulting in glucose uptake. We compared insulin-stimulated glucose uptake in FAP-As and ASC-As. It was assessed through [^3^H]2-deoxy-d-glucose uptake. As expected, insulin induced a clear stimulation of glucose uptake in ASC-As (Figure 4a). However, glucose uptake remained unchanged after insulin treatment in FAP-As, suggesting that FAP-As are insulin-resistant adipocytes.

Quantitative RT-PCR experiments demonstrated that FAP-As and ASC-As express similar levels of insulin receptor (IR) and Glut4 mRNAs (Figure 4b). This ruled out the possibility that the difference in glucose uptake is linked to a modification in the expression of these genes.

Insulin-stimulated glucose uptake is one of the end points of the insulin-signaling pathway, which is controlled by a cascade of phosphorylation events. We used western blot to analyze the phosphorylation status of key molecules of the insulin-signaling pathway, that is, IR, IRS-1, Akt, 42/44 MAP kinases (MAPK; Figure 4c and d). Insulin did not modify the phosphorylation status of these proteins in FAP-As, whereas, as expected, insulin induced an increase in the phosphorylation of the same proteins in ASC-As, revealing the activation of the insulin-signaling pathway in ASC-As. These differences between FAP-As and ASC-As observed in protein phosphorylation paralleled the differences found for glucose uptake. In FAP-As, the total inhibition of IR tyrosine phosphorylation, which is a very early insulin-signaling event, could explain why all subsequent steps are blunted, from IRS-1 tyrosine phosphorylations to glucose transport.

Protein tyrosine phosphatases are negative regulators of insulin signaling.^32^ So far, PTP1B and SHP2, two protein tyrosine phosphatases, have been proposed to act on IR and IRS-1. Their mRNA contents were examined by quantitative RT-PCR. As shown in Figure 4e and f, no significant difference was found between FAP-As and ASC-As. Dysregulation of these protein tyrosine phosphatases does not seem to be involved in FAP-As insulin insensitivity for glucose uptake.

Together, these results show that glucose uptake in FAP-As is insensitive to insulin and associated with a lack of insulin induction for key phosphorylations in the insulin-signaling pathway.

## Discussion

Adipose tissue is spread in different compartments and one of them can be found inside muscle tissue, as fatty infiltrations between muscle fibers bundles, and constitutes the so-called IMAT. This very particular localization and therefore the intimate contact between adipocytes and muscle fibers is likely crucial for muscle homeostasis. Nevertheless, the specificity for intermuscular adipocytes is poorly documented. A few studies have been performed in cattle, but no studies have been reported in humans to our knowledge. The recently identified adipogenic progenitors resident in skeletal muscle are the main IMAT contributors, considering their localization, proliferative capacities, abundance, and adipogenic potential after engraftment in muscle.^20, 21, 22^ Our study was conducted in humans regarding the potential clinical impact of IMAT for muscle homeostasis and repair. We found that FAP-As exhibited key cellular and functional properties of adipocytes derived from subcutaneous adipose tissue, but interestingly they also had their own specificities.

A first point is that the CD15^+^CD56^−^ progenitors that we previously identified and the PDGFR*α*^+^CD56^−^ progenitors identified by Uezumi *et al.*^24^ constitute the same fibro/adipogenic cell population. Our results fully confirm that the CD15^+^CD56^−^ immunophenotype is highly specific to human muscle FAPs. CD15 and PDGFR*α* should be considered as two unrelated FAP markers. CD15 is expressed on a variety of glycoproteins, glycolipids, and proteoglycans, and there are no available data on a possible co-localization with PDGFR*α*. Thus, both CD15^+^CD56^−^ and PDGFR*α*^+^CD56^−^ immunophenotypes allow prospective *in vitro* isolation of human FAPs. Muscle and adipose tissue progenitor cells could differentiate in the same culture medium, indicating close if not similar biochemical requirements. They followed similar differentiation kinetics and gave rise to adipocytes with very close phenotypes. FAP-As are smaller than ASC-As, and *in vivo* IMAT adipocytes are smaller than adipocytes of subcutaneous depots.^33, 34, 35^ Canonical early and late molecular markers of differentiation described in the adipose lineage exhibited similar profiles in muscle-differentiating progenitors. This indicates that the same stimulating molecular regulation of adipogenesis is shared by the fat muscle compartment, particularly regarding the expression of the key gene *PPARγ*. Furthermore, our data on adipogenesis inhibition extend this similarity to the negative regulation by TNF*α* and TGF*β* family. Moreover, the same level of expression of mature adipocyte markers such as FABP4 or adipsin strongly suggests that FAP-As and ASC-As have similar phenotypes. This is in agreement with FAP-As and ASC-As general appearances, characterized by the presence of multiple lipid droplets in cytosol. At a functional level, no significant differences were observed between FAP-As and ASC-As for the experimental measurement of triglycerides synthesis, content, and degradation. In addition, the impact of aging, which was found to lower triglyceride synthesis and degradation values in FAP-As, is in line with age-related changes described for ASC-As.^36^ Thus, the values of crucial basic functions in FAP-As are very close to those found for ASC-As. Together, our molecular, cellular, and biochemical results show that FAP-As are *bona fide* adipocytes.

Brown adipocytes have been largely described in rodents^37^ and also more recently in humans.^38^ In addition, ectopic brown adipocytes have been found in mouse skeletal muscle.^39^ The very low level of UCP1 detected with some FAP biopsies might be an insignificant signal or due to contamination by scarce brown adipocyte progenitors. Such progenitors can be scattered in white adipose tissue, which could have been mixed with muscle biopsies^40^ or they can be residents in skeletal muscle, although brown adipocytes progenitors have mainly been described in primary cultures derived from human fetus biopsies.^29^ We concluded that the muscle lineage investigated in this study is a white adipocyte lineage.

The other main finding is that FAP-As have a specific singularity. Unlike classical adipocytes, FAP-As were not sensitive to insulin in our *in vitro* experiments. This cannot be explained by an insufficient amount of insulin in our assays as it was used at high concentration (100 nM). The exact underlying molecular mechanisms of insulin resistance are still unclear. Here we show that the insensitivity to insulin is not mediated by altered expression of Glut4 or IR. Therefore, we investigated insulin signaling and found that the first steps of the pathway were modified. The addition of insulin failed to increase tyrosine phosphorylation of IR itself and consistently the cellular substrate IRS-1 did not undergo tyrosine phosphorylation. No insulin signal could thus be transmitted inside muscle adipocytes, as shown by the very weak induction of phosphorylation of Akt on threonine 308, one of the phosphorylations implying activation of this crucial protein kinase. The noninduction of phosphorylation of 42/44 MAPK by insulin confirms the impairment of insulin signaling and suggests that insulin-dependent pathways other than the regulation of glucose uptake, such as proliferation or survival, may be disturbed.

We did not find the reason for the lack of induction of IR and IRS-1 phosphorylations, but we can rule out dephosphorylations of these proteins that would be mediated by upregulations of the two important tyrosine phosphatases PTP1B and SHP2. Alternative mechanisms remain to be identified to account for the insulin-resistant status of FAP-As.

Only a few recent studies document relationships between IMAT accumulation and insulin resistance. They all conclude on a positive correlation between these two parameters.^41, 42, 43, 44^ However, there are no reports on the status of insulin sensitivity of IMAT in healthy subjects. We cannot definitely rule out that FAP-As insulin resistance was caused by our *in vitro* culture conditions. However, this would unlikely imply that the culture conditions would have different impacts on FAP-As and ASC-As, which were found to be very close regarding the other investigated parameters. Therefore our data strongly suggest that IMAT could be a very particular fat compartment, insensitive to insulin glucose uptake. This original feature opens the way for new exciting investigations. The main insulin-targeted tissues are liver, skeletal muscle, and fat tissue. A first point to consider is that IMAT is a minor fat compartment in healthy individuals and therefore its impact on regulation of circulating glucose is likely insignificant. A second point is the particular location of IMAT. It is the only fat compartment intimately bound to skeletal muscle. Many cross talks have been described in these two tissues, particularly in relation with the energy consumption of muscle and the energy storage of adipose tissue. It is thought that excess of fatty acids, that is, a basic fuel for muscle fibers, is stored in adipocytes to prevent cellular deleterious effects. The proximity of adipocytes should be advantageous for muscle regarding lipid storage and it is essential that IMAT fulfills this function. However, as muscle fibers, which represent a much greater tissue mass in healthy conditions than the associated IMAT, are endowed with insulin sensitivity for glucose uptake, we can assume that they are sufficient to absorb excess glucose from the local circulation, which brings blood to both muscle and intramuscular fat cells. Insulin sensitivity for IMAT would be insignificant because of its scarcity and its embedding in muscle tissue. This might be different in obese, immobilized, or aged individuals with a less favorable IMAT/muscle mass ratio, with the risk of exacerbating insulin resistance. It can also be speculated that the insulin resistance of IMAT would prevent local competition between muscle and IMAT for glucose uptake. Glucose would thus be directed mainly toward muscle for its energy needs.

FAPs have remarkable properties. They are mesenchymal progenitors^23^ also endowed with a fibrogenic potential. In addition, they are important actors of muscle repair as they activate differentiation of satellite cells, which in turn inhibit differentiation of FAPs into adipocytes under healthy conditions.^21, 25, 45^ No equivalent population has been described in adipose tissue compartments. It has recently been shown that adipogenic progenitors from subcutaneous adipose tissue can also differentiate into fibrogenic cells.^46^ However, stimulation of the myogenic potential of satellite cells, as well as the inhibition of differentiation into adipocyte by satellite cells, are restricted to muscle FAPs and were not found with adipogenic progenitors from at least subcutaneous adipose tissues (unpublished observations). Thus, unlike other adipogenic progenitors, FAPs show specific cross talks with myogenic progenitors resident in skeletal muscle and give rise to adipocytes that are independent of insulin for their glucose uptake. Muscle adipogenic lineage has thus specific impacts on muscle physiology at both levels of undifferentiated progenitors and terminally differentiated adipocytes. At this point, we do not have enough clues to speculate on relationships between the resistance to insulin signaling and interactions with myogenic progenitors. An indication would come from the comparison of muscle adipogenic lineage properties under healthy and pathophysiological conditions. There is no more inhibition of adipogenic differentiation of FAPs in aging (unpublished observations) and likely in muscle dystrophies, as fat infiltrates muscle fibers. In addition, in aging, FAPs lose their capacity to support myogenic differentiation of satellite cells.^47^ Investigations on the status of insulin resistance of FAP-As under these pathophysiological conditions would reveal whether the impairment of FAP cross talks with satellite cells is linked to the lack of FAP-As' insulin sensitivity.

In conclusion, FAP-As exhibit several canonical features of ASC-As with the notable exception of a lack of sensitivity to insulin. Therefore, FAP cells recently identified in mouse and human skeletal muscle, and isolable on the basis of specific cell-surface markers by flow cytometry, could give rise to a genuine but specific fat compartment. This potential sheds new light on muscle homeostasis and cross talks between adipose and muscle tissues.

## Materials and Methods

### Progenitor cells

Tissue samples were obtained as *res nullius* from surgeries on healthy donors without obesity or diabetes. All protocols were approved by the Centre Hospitalier Universitaire de Nice Review Board, according to the rules of the French Regulatory Health Authorities. Biopsies were obtained from 15 muscle donors and 5 adipose tissue donors (Table 1). Biopsies from age-matched donors under 9 years old were used for all experiments. In addition, adult donor muscle specimens were used when indicated.

Skeletal muscle cells were isolated by a standard method.^48^ Briefly, skeletal muscle was minced into 1 mm^3^ fragments and digested at 37 °C, first using Liberase (Roche Diagnostics, Meylan, France, http://roche.fr/portal/eipf/france/rochefr/recherche/home) for 1 h and then using 0.25% trypsin-EDTA (Lonza Verviers, Verviers, Belgium) for 20 min. The cell suspension was homogenized, filtered through 40-*μ*m cell strainers (BD Biosciences, Le Pont de Claix, France), and plated in growth culture medium.

For ASCs, about 200 mg/ml of adipose tissue was dissociated for 10–20 min in Dulbecco's modified Eagle's medium (DMEM) containing 100 U/ml penicillin and 100 *μ*g/ml streptomycin, 2 mg/ml collagenase A, and 20 mg/ml BSA. The crude stromal vascular fraction was separated by low speed centrifugation (200 *g*, 10 min). The adipocyte fraction was discarded and pelleted cells were seeded at 1000–3500 cells/cm^2^ in growth culture medium.

### Cell culture

Cell culture media, serum, buffer, and trypsin were purchased from Lonza Verviers (http://www.lonza.com/group/en/company/sites/europe/lonza_verviers_sprl.html) and cell culture reagents from Sigma-Aldrich Chimie (Saint-Quentin Fallavier, France, http://www.sigmaaldrich.com/france/contactez-nous.html).

The growth culture medium for muscle progenitor cells was Ham's F10 medium supplemented with 20% fetal bovine serum. The growth culture medium for adipose tissue progenitors was low-glucose DMEM supplemented with 10% fetal bovine serum. The two media were complemented with 10 mM Hepes, 10^−6^ M dexamethasone, 2.5 ng/ml basic fibroblast growth factor, 100 U/ml penicillin, and 100 mg/ml streptomycin.

Adipogenic differentiation was induced 2 days after confluence (designated as day 0) by switching to DMEM/Ham's F12 50/50 (v/v) complemented with 1 *μ*M dexamethasone, 0.1 mM 1-methyl-3-isobutylmethyl-xanthine (MIX), 860 nM insulin, 10 *μ*g/ml transferrin, 0.2 nM triiodothyronine, and 100 nM rosiglitazone PPAR*γ* gonist). Three days later, cells were placed in the same medium lacking MIX and dexamethasone. Cells were studied after 2 weeks of adipogenic differentiation, except when indicated. Myogenic differentiation was induced at confluence in Ham's F10 complemented with 10 *μ*g/ml insulin, 5 *μ*g/ml transferrin, and 2% horse serum. Fibrogenic differentiation was done in Ham's F10 complemented with 1% horse serum and 5 ng/ml TGF*β*-1.

### Fluorescence analyses

They were performed as previously described.^18^ Immunofluorescence analyses of adherent cells were carried out with antimyosin heavy chains (Developmental Studies Hybridoma Bank) and anti-*α* smooth muscle actin (Sigma-Aldrich) antibodies. Living cells were sorted by flow cytometry according to labeling with anti-CD56-APC, anti-CD15-FITC, and CD140a-PE (PDGFR*α*) antibodies (BD Biosciences). We used a BD FACSARIA II sorter equipped with four lasers (Becton, Dickinson and Company, Franklin Lakes, NJ, USA, http://www.bdbiosciences.com/us/s/contactus) and the BD FACSDiva software. For CD15-FITC, fluorescence was excited with the 488 nm laser and measured with a 530/30 bandpass filter. For CD140a-PE, fluorescence was excited with the 561 nm laser and measured with a 586/15 bandpass filter. For CD56-APC, fluorescence was excited with the 633 nm laser and measured with a 670/14 bandpass filter. Fluorescence minus one (FMO) controls were performed to insure that the FITC fluorescence did not spread into the PE fluorescence (Supplementary Figure 1). The fluorescence spreads were always <1%. Gates created after compensation with the appropriate FMO controls were the same as those created with single-staining controls.

### Adipocyte morphometry analysis

Differentiated adipocytes were PBS-washed, fixed in 4% paraformaldehyde for 10 min, treated for 15 min with oil red O (60% of a stock solution at 0.5% w/v in isopropanol and 40% distilled water), and counterstained with crystal violet. Images were recorded with a TE-2000U bright-field optical microscope (Nikon, Tokyo, Japan). Cell and lipid droplet areas were analyzed using ImageJ software (National Institutes of Health, Bethesda, MD, USA). At least 300 adipocytes from each sample were measured. Areas were measured in *μ*m^2^ by the software. The amount of lipid droplets was counted per cell. Incomplete droplets located at the edge of the image were excluded.

### Gene expression analysis

Total RNA was extracted using TRI-reagent (Euromedex, Souffelweyersheim, France, http://www.euromedex.com/). RNA was treated with DNase I (Promega, Charbonnieres, France) for 30 min before reverse transcription. First-strand cDNA was generated on 1 μg of RNA with Moloney murine leukemia virus reverse transcriptase (Promega) in the presence of 12.5 ng/l random primers for 1 h at 37 °C. Integrity of cDNA was checked by amplification of 18S rRNA with classical PCR. For quantitative PCR (qPCR), the final reaction volume was 10 *μ*l including specific primers (0.3 *μ*M), 8 ng reverse-transcribed RNA, and 10 *μ*l SYBR Green Master Mix (Ozyme, Montigny Le Bretonneux, France). The qPCR conditions were as follows: 2 min at 50 °C, 10 min at 95 °C, followed by 40 cycles of 15 s at 95 °C, 1 min at 60 °C. qPCR assays were run on a StepOneplus Real-time PCR system (Applied Biosystems, Courtaboeuf, France). Quantification was performed using the comparative-ΔCt method. The housekeeping gene TATA box-binding protein was used as reference. All primer sequences were designed using Primer Express software (Applied Biosystems). Primers were validated by testing the PCR efficiency using standard curves (85%<efficiency<115%). The 5′–3′ sequences of forward and reverse primers were, respectively, GTTGGTGGAGCGATTTGTCT and GGCCTCACTAAACCATCCAA for 18S RNA, CTGCGTGGCTGGTGATGAG and CAGGTCGTCTCCACCCTTGA for MCK, TGCCTGCATGGGCAAGTGA and CTGGGCAGCGGAAACG for *α*SMA, AGCCTCATGAAGAGCCTTCCA and TCCGGAAGAAACCCTTGCA for PPAR*γ*, ATGGGATGGAAAATCAACCA and TGCTTGCTAAATCAGGGAAAA for FABP4, AACCAACCGCACATGCAGAT and GGCAGAGGGAGAAGCAGAGAGT for CEBP*β*, AGGGAGACCGAGCGCTTTC and TGCATCTCCACACACCAAACC for leptin, GGGAAAGTCACTGCGACATGAT and ACGTCGGATTCAAATACAGCATAGA for CD36, AGGGTCACCCAAGCAACAAAG and TACGTGGCCCATGCTGATCT for adipsin, GCAGTCTGTGGTTCTGATTCCATAC and GCCCTTGAGTCGTGGTTTCC for adiponectin, TGGAGGTACTTTTCAGCCAGGAT and TCGTGGGAGCACTTCACTAGCT for LPL, CATTCCTTGGTTCATCGTG and ATAGCCTCCGCAACATAC for Glut4, TGCTGCTCCTGTCCAAAGAC and GAGATGGCCTGGGGACGAAA for IR, GTGTGCCCAACTGTGCAATG and CCAGGATCCAAGTCGCAAGA for UCP1, TGA TCC AGA CAG CCG ACC A and ATG AAT TTG GCA CCT TCG for PTP1B, GACTTTTGGCGGATGGTGTTCC and CGGCGCTTTCTTTGACGTTCCT for SHP2, and CACGAACCACGGCACTGATT and TTTTCTTGCTGCCAGTCTGGAC for TBP.

### Western blots

Adipocytes were deprived of insulin for 72 h in DMEM/Hams' F12 50/50 (v/v) complemented with 0.2% BSA and then stimulated for 20 min with 100 nM insulin. They were collected with a rubber policeman in 200 *μ*l RIPA buffer (150 mM NaCl, 1% Nonidet P-40, 0.5% sodium deoxycholate, 0.1% SDS, 5 mM NaF, 2.5 mM Na_4_P_2_O_7_, 2 mM sodium vanadate, 5 mM EDTA, protease inhibitor cocktail (Roche Diagnostics), and 50 mM Tris-HCl, pH 8.0). The protein content was determined according the BCA method (Pierce BCA Protein Assay Kit, Thermoscientific, Rockford, IL, USA; #23227).

Equal amounts of protein were resolved by 7.5% SDS-PAGE under reducing conditions and transferred to Immobilon–P membranes (Millipore Corporation, Bedford, MA, USA). A unit of 20 *μ*g of proteins was used for insulin-signaling measurements. For immunoblotting assays, the primary antibodies were mouse anti-phosphotyrosine (Upstate, Hertfordshire, UK), rabbit anti-phospho-Akt (Thr308; Ozyme), rabbit anti-Akt (Ozyme), mouse anti-phospho-p44/42 MAPK (Thr202/Tyr204) and mouse anti-*β*-tubulin (Sigma-Aldrich). The bound primary antibody was detected by horseradish peroxidase-conjugated secondary antibody (Promega) and visualized with an electrochemical luminescence detection kit (Millipore). Chemiluminescence was observed and quantified using a ChemiDoc XRS plus (Bio-Rad, Marnes-la-Coquette, France). The band intensity was measured using the Quantity One software (Bio-Rad).

### GPDH activity, lipolysis, triglyceride content, and glucose uptake

GPDH activity was measured as described previously^49^ and expressed in mU/mg of protein.

Lipolysis was assessed by measuring glycerol release from differentiated cells as follows: after 72 h of insulin depletion, the cells were maintained in 2% BSA for 3 h of incubation at 37 °C in the presence or absence of 10 *μ*M forskolin; glycerol assays were then performed.

For the measurement of triglyceride content, cells were washed twice with PBS and then lysed with 5% NP40 in water. Glycerol and triglyceride were quantified using Free Glycerol Reagent (Sigma, Lyon, France; F6428) and the Triglyceride Quantification Kit (Biovision, Mountain View, CA, USA; #K622-100) according to the manufacturers' instructions. The protein concentration was determined with the Pierce BCA Protein Assay Kit (#23227).

Insulin-stimulated glucose transport was determined by measuring the amount of nonmetabolizable [^3^H]2-deoxy-d-glucose transported into adipocytes, as already described.^50^ After overnight serum depletion, fully differentiated adipocytes were washed with Krebs–Ringer phosphate buffer (10 mM phosphate buffer, pH 7.4, 1.25 mM MgSO_4_, 1.25 mM CaCl_2_, 136 mM NaCl, and 4.7 mM KCl) and incubated without or with insulin (100 nM) for 20 min in Krebs–Ringer phosphate buffer supplemented with 0.2% BSA. Identical levels of adipogenic differentiation with or without insulin treatment were validated with ImageJ software analysis of total adipocyte areas on culture well pictures. Glucose transport was determined by the addition of 2-[3H] deoxyglucose (0.1 mM, 0.5 *μ*Ci/ml). The reaction was stopped after 3 min at 37 °C by washing the cells with ice-cold PBS. Cells were lysed in RIPA buffer and glucose uptake was assessed by scintillation counting. The results were normalized for protein content measured by BCA assay.

### Statistical analysis

Differences between data groups were evaluated for significance using the two-tailed unpaired Student's *t-*test. A *P-*value <0.05 was considered significant. The data are presented as mean±S.E. of the mean of independent measurements. The number of measurements is indicated in figure legends.

## Figures and Tables

**Table 1:** Muscle and adipose tissue biopsies

**Biopsy reference**	**Gender of donor**	**Age of donor**	**Origin**
*ASCs*			
ASCs 1	Male	9 years	Subcutaneous
ASCs 2	Male	9 years	Subcutaneous
ASCs 3	Male	6.5 years	Subcutaneous
ASCs 4	Female	19 months	Subcutaneous
ASCs 5	Male	4 months	Subcutaneous
			
*FAPs*			
FAPs 1	Female	1 year	Quadriceps
FAPs 2	Male	1 year	Gluteus maximus
FAPs 3	Female	1 year	Abdominal
FAPs 4	Male	14 months	Inguinal
FAPs 5	Male	21 months	Inguinal
FAPs 6	Female	2 years	Inguinal
FAPs 7	Male	2 years	Abdominal
FAPs 8	Male	19 months	Inguinal
FAPs 9	Male	4 years	Abdominal
FAPs 10	Male	8 years	Inguinal
FAPs 11	Male	14 years	Deltoid
FAPs 12	Female	17 years	Gluteus maximus
FAPs 13	Male	46 years	Vastus lateralis
FAPs 14	Female	51 years	Gluteus medius
FAPs 15	Male	59 years	Quadriceps

## Abbreviations

IMAT: intermuscular adipose tissue
FAP-As: fibro/adipogenic progenitor-derived adipocytes
ASC-As: adipose stroma cell-derived adipocytes
