# Supplementary figures and images for: Characterization of adipocytes derived from fibro/adipogenic progenitors resident in human skeletal muscle
Source: Cell Death Dis. 2015 Apr 23;6(4):e1733–. doi: 10.1038/cddis.2015.79 (PMC4650547; doi:10.1038/cddis.2015.79)

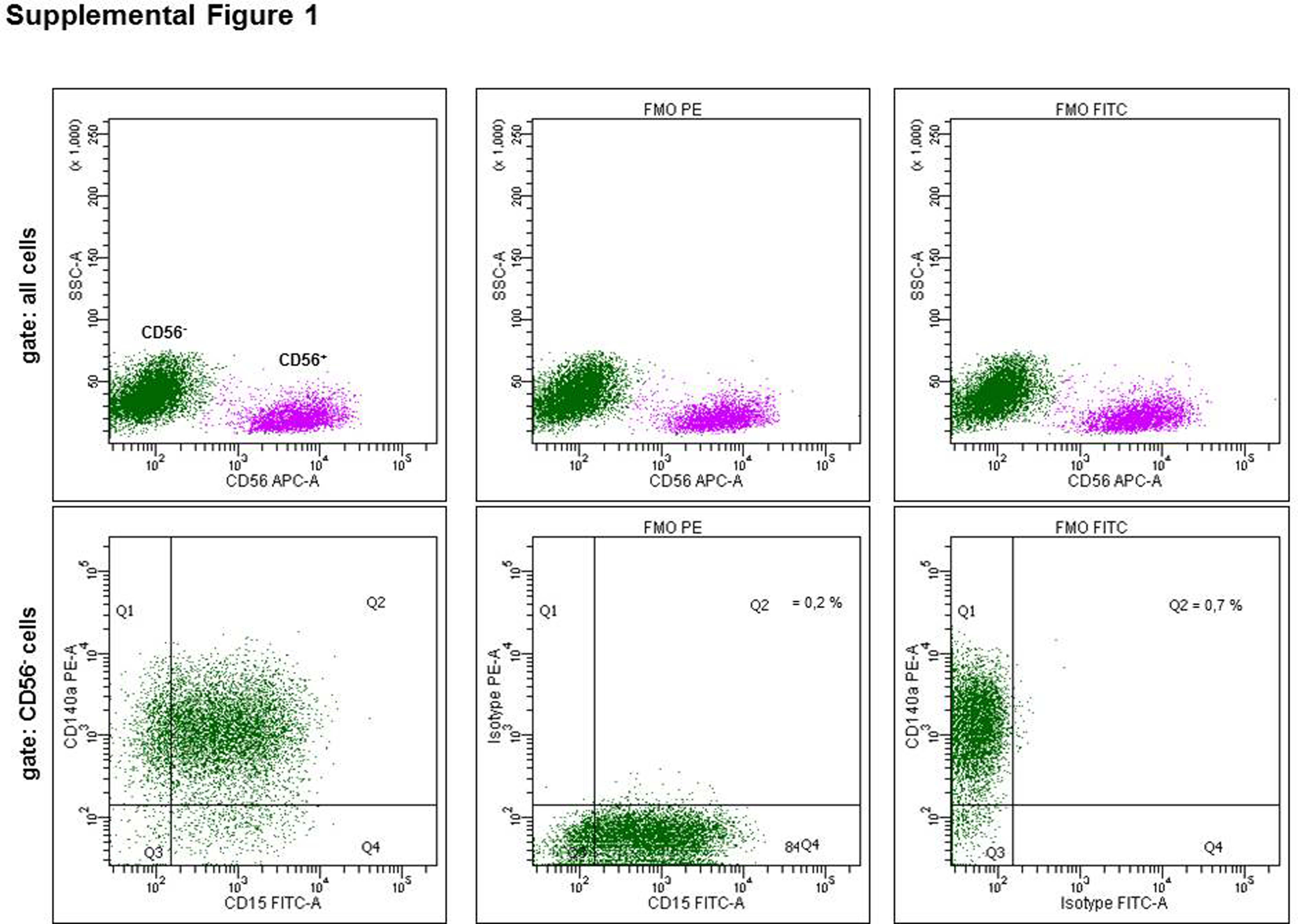

Supplement: Supplementary Figure 1 [file cddis201579x1.tif]

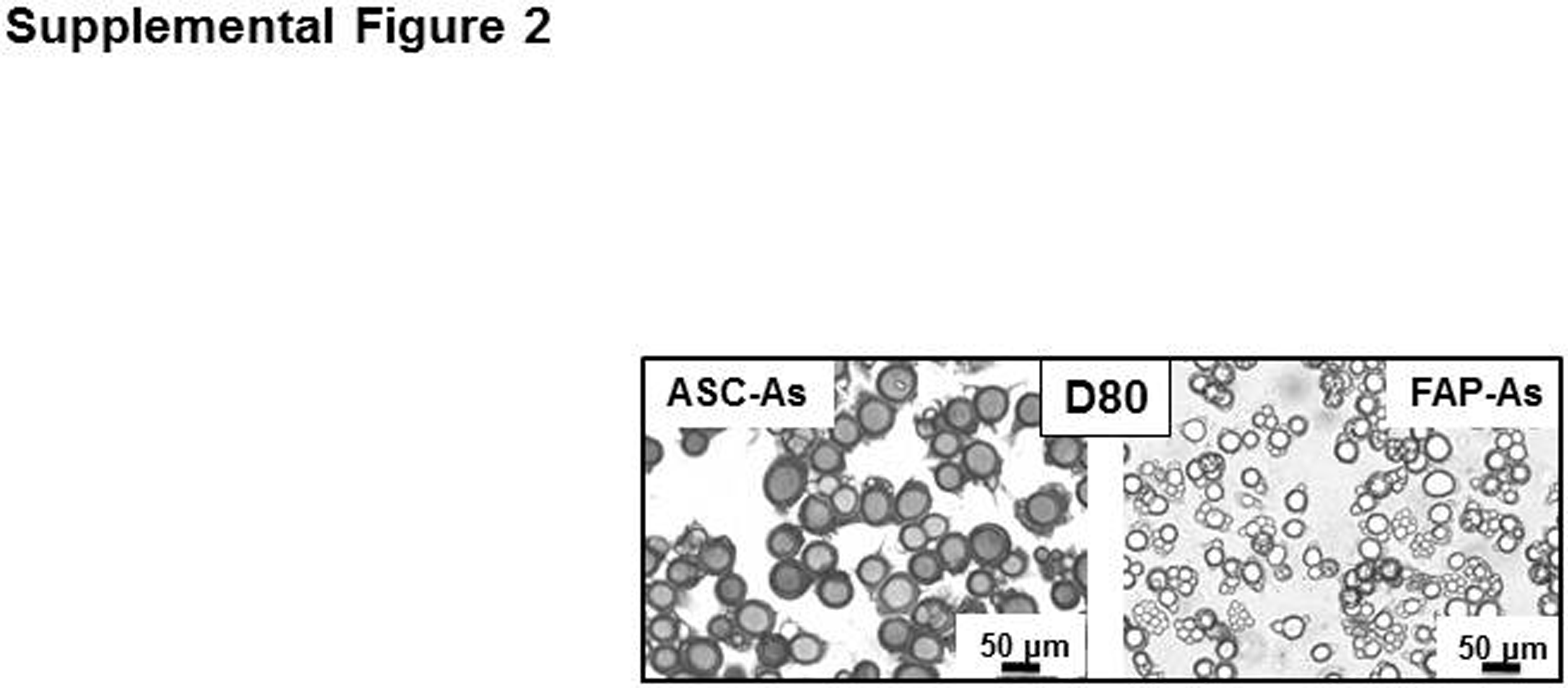

Supplement: Supplementary Figure 2 [file cddis201579x2.tif]

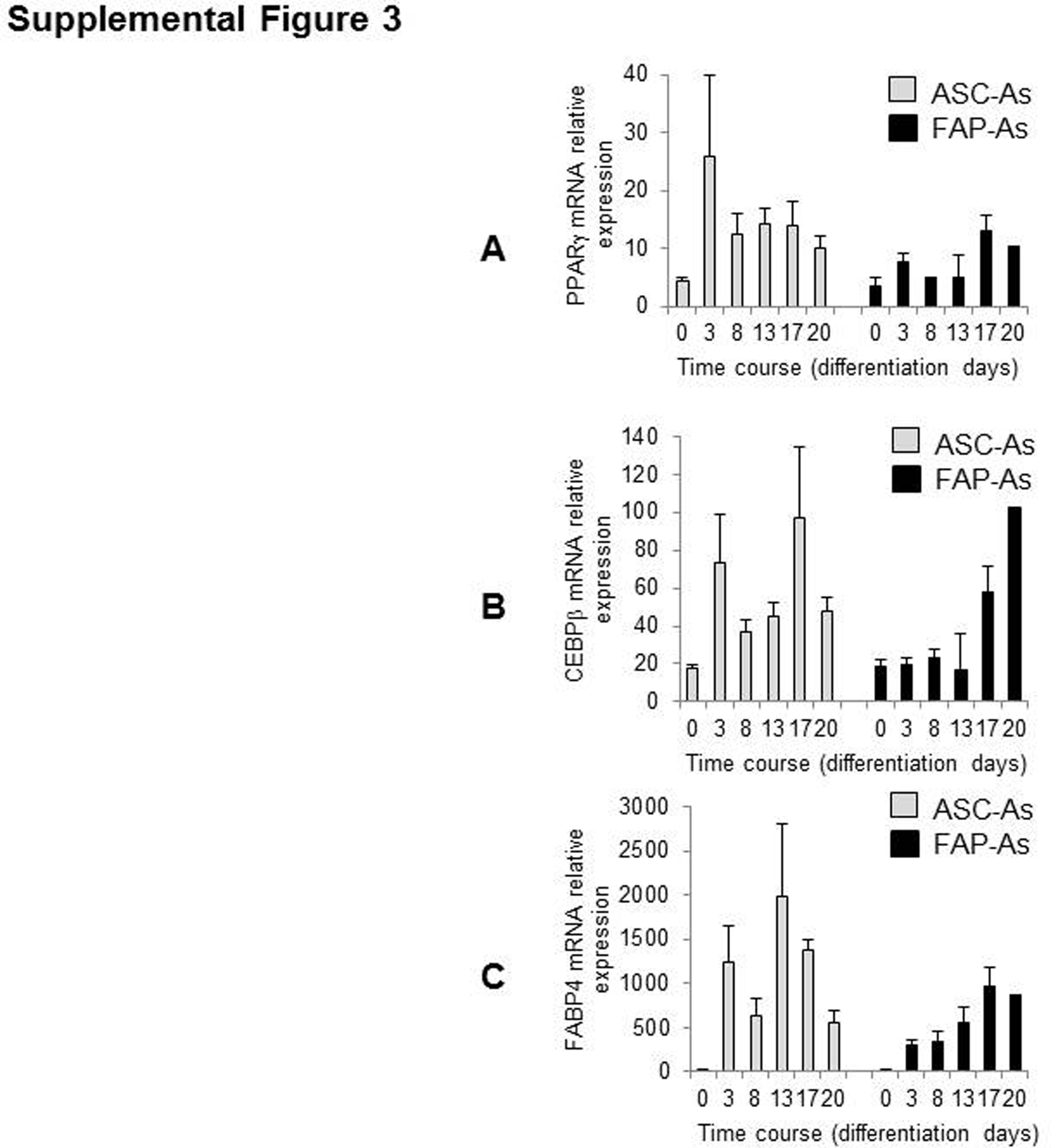

Supplement: Supplementary Figure 3 [file cddis201579x3.tif]

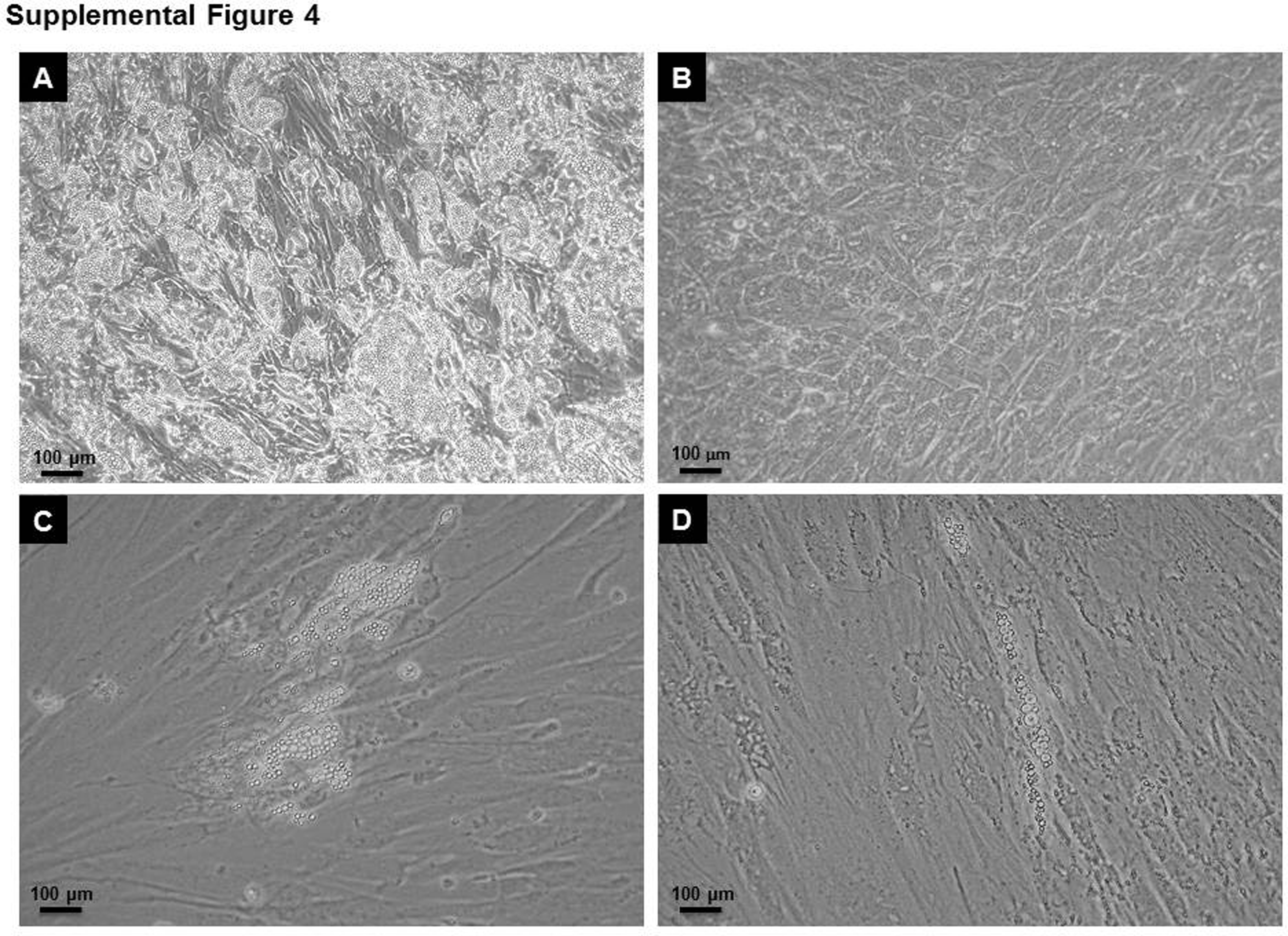

Supplement: Supplementary Figure 4 [file cddis201579x4.tif]
