# Supplementary material for: Characterization of adipocytes derived from fibro/adipogenic progenitors resident in human skeletal muscle
Source: Cell Death Dis. 2015 Apr 23;6(4):e1733–. doi: 10.1038/cddis.2015.79 (PMC4650547; doi:10.1038/cddis.2015.79)
Supplement: Supplementary Figure Legends [file cddis201579x5.doc]

**Supplemental Figure 1. Fluorescence minus one (FMO) controls**

The data were obtained with the representative FAPs biopsy 2. First column : the CD56^-^ cells (upper panel) were analyzed with both anti-CD15-FITC and anti-PDGFRα (CD140a)-PE (lower panel). Second column: CD140a-PE was replaced by the appropriate anti-IgG2aκ-PE isotype as negative control. Third column: CD15-FITC was replaced by the appropriate anti-IgM κ-FITC isotype as negative control. Percentages of unspecific fluorescence are indicated in quadrants 2.

**Supplemental Figure 2. Long term adipocyte differentiation**

FAP-As and ASC-As were kept in differentiation-inducing medium during 80 days. In the left panel cells were stained with Oil Red O. Pictures were visualized by light microscopy with x 200 magnification. Representative fields obtained with ASC biopsy 4 and FAP biopsy 6 are shown.

**Supplemental Figure 3. Time course of adipogenic markers expression**

PPARγ **(A)**, CEBPβ **(B)**, and FABP4 **(C)** relative mRNA expressions were monitored by quantitative RT-PCR in FAP-As (n = 3; FAP biopsies 11, 14, and 15) and ASC-As (n = 3; ASC biopsies 2, 3, and 4) at days 0 (switch to differentiation-inducing medium), 3, 8, 17, and 20. No significant differences were found between FAP-As and ASC-As. The results are mean ± standard error of the mean for 3 independent measurements.

**Supplemental Figure 4. Inhibition of adipogenic differentiation**

Muscle adipogenic progenitors were grown to confluence and then treated with differentiation-inducing medium for 7 days. Adjacent culture wells were compared for no cytokine treatment **(A)**, TGF-β1 (5 ng / ml) **(B)**, TNF-α (10 ng / ml) **(C)**, and activin A (100 ng/ml) **(D)**. Intracellular lipid droplets were assayed with Oil Red O staining and visualized by light microscopy with 100× magnification. Scale bar = 100 μm. Cells from the representative FAP biopsy 9 are shown.
